# Timely Shaver Treatment Removes Chronic Tophaceous Mass Improve Surgical Outcomes

**DOI:** 10.7150/ijms.95372

**Published:** 2024-07-09

**Authors:** Patrick Szu-Ying Yen, Hung-Pin Tu, Shu-Hung Huang, Su-Shin Lee

**Affiliations:** 1 School of Medicine, Kaohsiung Medical University, Kaohsiung, Taiwan.; 2 Department of Public Health and Environmental Medicine, School of Medicine, College of Medicine, Kaohsiung Medical University, Taiwan.; 3 Division of Plastic Surgery, Department of Surgery, Kaohsiung Medical University Hospital, Kaohsiung, Taiwan.; 4 Regenerative medicine and cell therapy research center, Kaohsiung Medical University, Kaohsiung, Taiwan.; 5 Department of Surgery, Faculty of Medicine, College of Medicine, Kaohsiung Medical University, Kaohsiung, Taiwan.; 6 Department of Surgery, Kaohsiung Municipal Siaogang Hospital, Kaohsiung, Taiwan.

**Keywords:** shaver, tophus, gout, surgical complication

## Abstract

**Background:** Current treatments with urate-lowering therapy (ULT) are effective for most patients with gout. However, approximately 10% of these patients do not respond well to ULT and develop chronic tophus lesions.

**Objective:** This study aimed to evaluate the efficacy of surgery involving the shaver technique against chronic tophus lesions.

**Methods:** This single-center, retrospective cohort study included 217 patients who had cumulatively undergone 303 shaver-assisted procedures between 2002 and 2018. Surgical outcomes were assessed in terms of the length of hospital stay (LOS) and wound healing time.

**Results:** LOS and wound healing time were longer in patients with a preoperative tophus infection and lower extremity lesions than in those without infection and with upper extremity lesions (respectively, LOS: 12.7 vs. 8.6 days; wound healing time: 22.7 vs. 16.3 days). However, factors such as age, sex, body mass index, renal function, or uricemia level exerted no significant effect on surgical outcomes.

**Conclusion:** Surgery involving the shaver technique should be performed before tophus infection. Clinical outcomes tend to be better for upper extremity lesions than for lower extremity lesions.

## Introduction

Gout is a modern disease caused by the deposition of monosodium urate crystals in joint spaces. Risk factors for tophaceous gout include race, sex, age, central obesity, and family history 1,2. Clinically, gout manifests as recurrent acute attacks with severe pain or as chronic inflammation with tophaceous deposits in joints 3. Urate-lowering therapy (ULT) is a first-line treatment for gout 4. ULT reduces the serum level of urate over a prolonged period to achieve permanent remission of gouty arthritis. Notably, approximately 10% of all patients receiving ULT do not respond to this therapy 5. For these patients, conventional surgical intervention is necessary in cases of ulceration or wound infection 6. However, conventional surgery carries a high risk of complications and may disrupt blood circulation, causing necrosis of the overlying skin 7,8.

To mitigate the risks associated with conventional surgery and treat patients who do not respond to ULT, Lee et al. 1 proposed the soft tissue shaver technique 9, which facilitates early surgical intervention for patients with chronic gout. The present study was conducted to evaluate the efficacy of surgery involving the shaver technique against chronic tophus lesions.

This study included 217 patients who had undergone shaver-assisted surgery, and 47 of them underwent multiple procedures. Of these 47 patients, 36 underwent consecutive surgery within one year. The shaver technique, in which one arm and one leg are operated at a time, allows patients to maintain partial self-care ability and some mobility. Patients often report satisfaction with this treatment because it does not burden their family members. Consequently, patients often refer this procedure to their relatives. This phenomenon reflects the hereditary nature and familial aggregation of tophus lesions.

## Methods

### Data collection

We analyzed data from 303 shaver procedures performed for chronic tophus lesions at Kaohsiung Medical University Hospital between March 2002 and December 2018. Patients with tophus lesions who had voluntarily undergone shaver-assisted surgery were included (n = 217) in this retrospective study. No exclusion criteria were set for patients willing to undergo shaver-assisted surgery. Informed consent was obtained from all patients before surgery.

### Surgical technique

**Figure 1:**
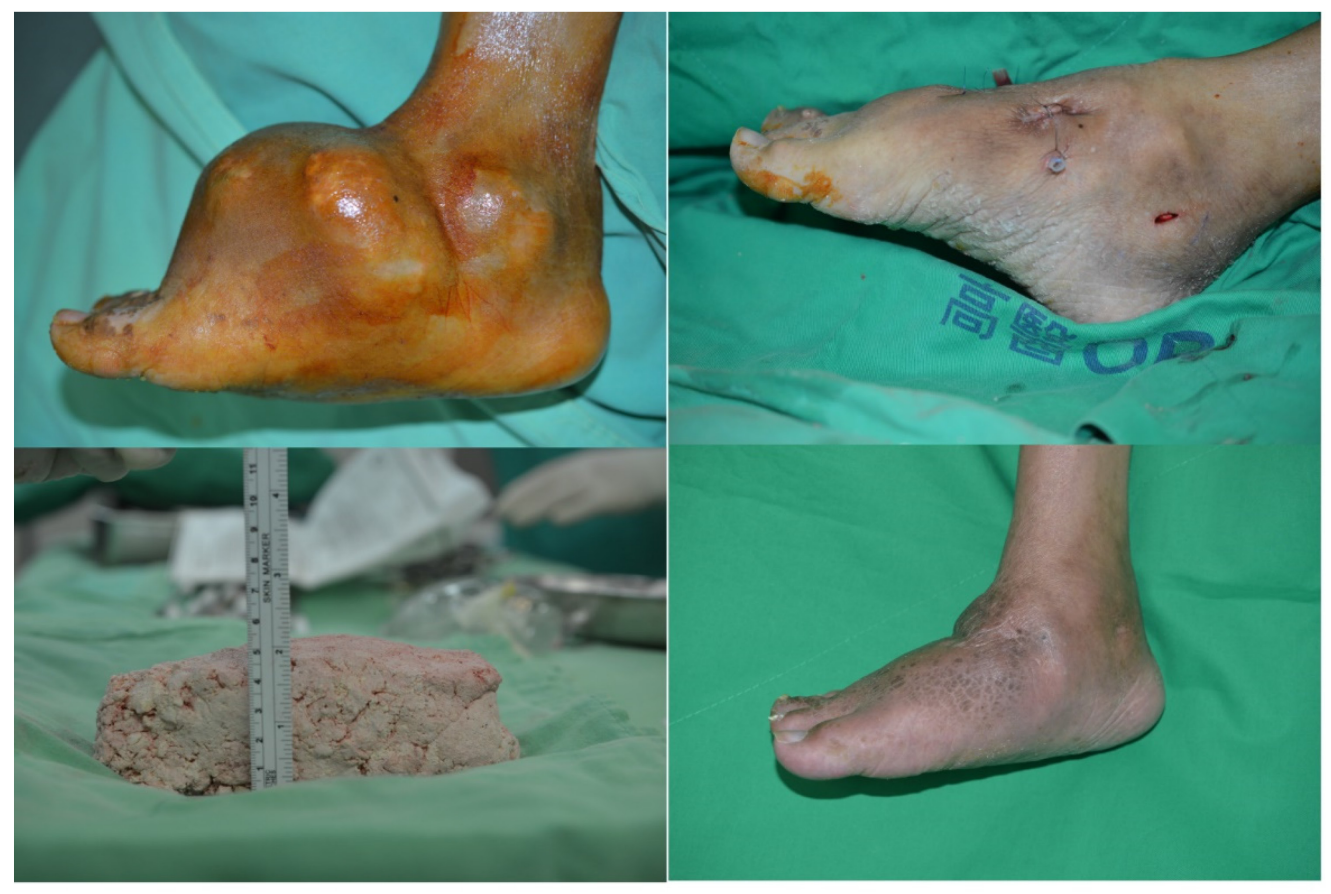
(Top left) Pre-surgery picture of the right foot, 55 year-old male patient, medial view; (Bottom left) Tophaceous gout removed; (Top right) Immediate post operation with penrose drainage of the same patient, medial view; (Bottom right) 11 months follow up.

**Figure 2:**
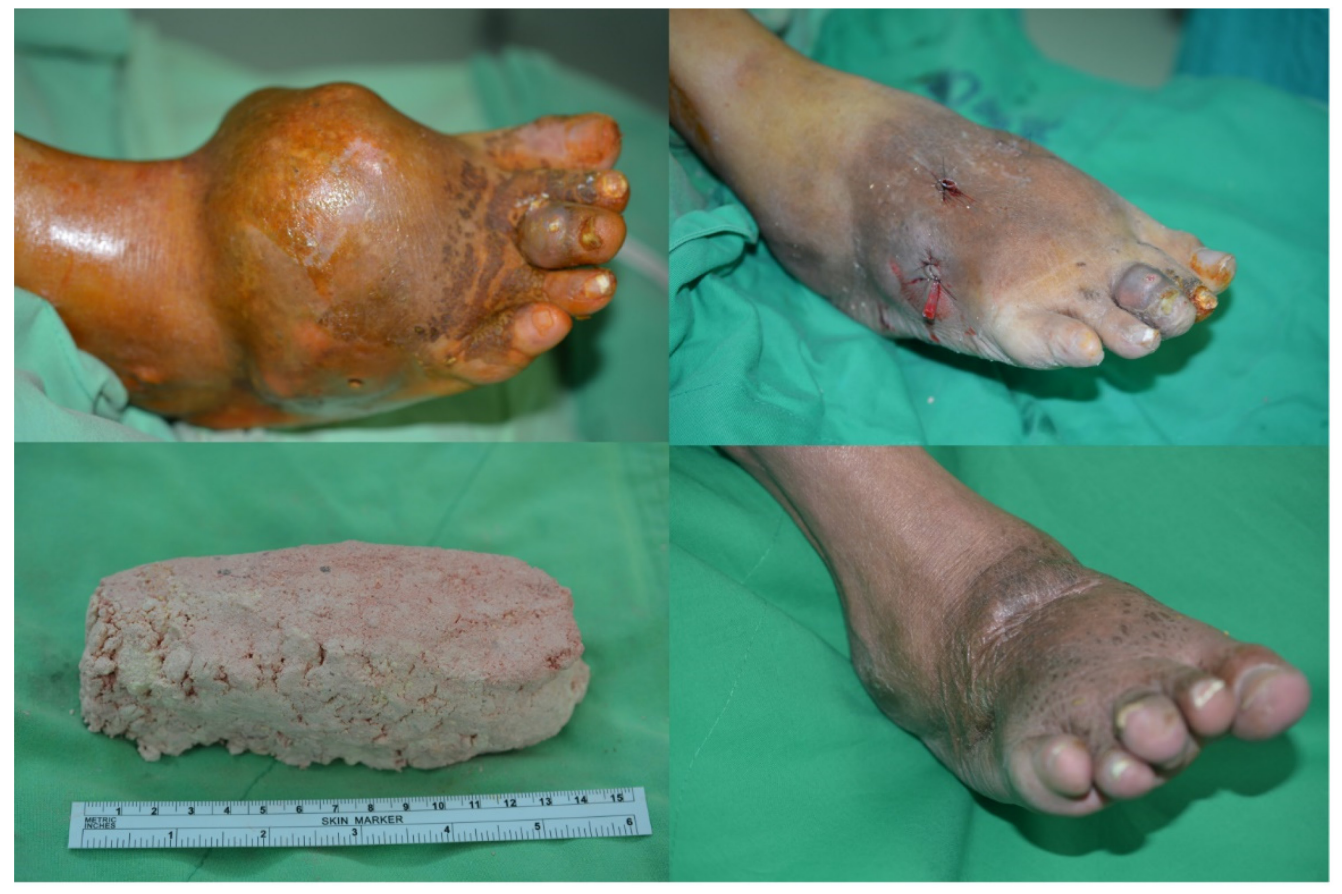
(Top left) Pre-surgery picture of the right foot, 55 year-old male patient, lateral view; (bottom left) Tophaceous gout removed; (Top right) Immediate post operation with penrose drainage of the same patient, lateral view; (Bottom right) 11 months follow up.

Surgery was performed using the powered shaver system (Conmed Linvatec; Largo, FL, USA). Warm normal saline solution (37°C) was added to the irrigation system to prevent obstruction of the shaver tip and increase the solubility of tophaceous materials; a two-fold increase was noted in the solubility of urate when the temperature reached 37°C compared with that when the temperature was 25°C 10. Before surgery, the vasculature of the skin overlying the tophus lesion was inspected to minimize accidental interruption of vessels. In addition, surgeons took precautions to avoid injury to underlying structures, such as the ulnar nerve at the elbow. A tourniquet was used to minimize intraoperative blood loss. During surgery, 5-mm incisions were made to facilitate access to each tophus lesion. After incision, a curette was inserted to remove chalky materials for pathological analysis and create a working cavity for the shaver. The oscillating inner blade, rotating at 3000 rpm, enabled precise debridement of the tophus lesion. Although the shaver can cut in a multidirectional fashion, constant bimanual palpation of the tophus lesion during shaving is required to prevent over-shaving of the skin envelope. For safety, relatively inexperienced surgeons should maintain the tip-side port upward to avoid causing trauma to surrounding structures. During surgery, surgeons can monitor blood loss by observing the color of the washout. After the removal of approximately 70%-80% of the tophus, warm saline was used to irrigate the tophus cavity, and manual curettage was performed to detect residual tophus particles (Figures 1 and 2). The shaver technique enabled the removal of a tophus lesion within 15 min per site.

In patients with multiple tophi, the infected tophus was treated last to reduce the risk of intraoperative contamination. When tophi were located around critical areas, such as the phalangeal joints, the shaver was used to break the tophus capsule; this was followed by manual curettage to minimize tendon damage. Furthermore, to minimize postoperative discomfort, one upper extremity and one lower extremity were operated during a given surgical session, thereby allowing the patient to have partial self-care ability in the postoperative period and facilitating early discharge.

After shaving and irrigation, Penrose drains should be inserted into these extremities. All of our patients received ULT on their first visit to our clinic. Most patients received medical treatment two weeks before surgery. Low-dose colchicine was prescribed to reduce pain and prevent acute gout attacks. Although the prescription of high-dose colchicine is relatively common in patients with acute gout, low-dose colchicine has been demonstrated to exhibit improved efficacy 11.

### Statistical analysis

The following data were collected from the hospital's electronic medical records: age, sex, surgery site, body mass index, surgery date, suture removal date, and medical information indicating whether the patient had a preoperative infection or renal impairment.

Although only 217 patients were included in this study, 303 procedures were performed. Given the varying recovery periods, each procedure was treated as a separate case.

Patients were categorized on the basis of at-admission blood reports and surgical sites. Patients were considered to have a preoperative infection if their white blood cell count exceeded 10,000/µL. Renal disease was categorized as normal if the patient's creatinine level was <1.3 mg/dL. Hyperuricemia was defined as a serum uric acid level of ≥7 mg/dL.

Patients were further stratified into three groups by the surgical site: upper extremity, lower extremity, or simultaneous surgery of one arm and one leg.

The length of hospital stay (LOS) was defined as the interval (number of days) from the date of surgery to that of discharge. Wound healing time was defined as the interval (number of days) between the date of surgery and that of suture removal. Shorter durations in both measures indicated a better prognosis.

Because of the prolonged follow-up period, the amount of available data varied across the patient groups (Tables 1-4).

An independent *t-test* was used for binary variables, and the one-way analysis of variance test was used for nominal variables. A *p-value* of <0.05 indicated statistical significance at a 95% confidence interval level. All statistical analyses were performed using SPSS (version 22; IBM Corporation, Armonk, NY, USA).

### Ethics

This study was approved by the Institutional Review Board of Kaohsiung Medical University Chung-Ho Memorial Hospital (protocol code: KMUHIRB-E(I)-20190343; approval date: 2020/1/6) and conducted in accordance with the ethical principles of the Declaration of Helsinki.

## Results

During the study period, 303 procedures were performed in 217 patients. Of the patients, 95% were men and 52% had a body mass index of 16-24 kg/m2. The patients were aged between 30 and 50 years. Hyperuricemia was detected in 74% of the patients, with 63% having no prior gout attacks. Most patients had no preoperative infection or renal impairment.

Surgical outcomes were measured in terms of the average LOS and wound healing time. The average LOS was 12.7 days for patients with a preoperative infection and 8.6 days for those without it. Furthermore, the average LOS was 11.1 days for patients with impaired renal function and 9.1 days for those with normal renal function. Patients with hyperuricemia had an average LOS of 10.76 days, whereas those without hyperuricemia had an average LOS of 10.04 days. The average LOS was 19.4 days for patients undergoing lower extremity surgery, 12.7 days for those undergoing upper extremity surgery, and 18.0 days for those undergoing surgery involving both upper and lower extremities.

Wound healing time varied across the patients. Patients with a preoperative infection had wounds that healed in 22.74 days, whereas those without it had wounds that healed in 16.3 days. Patients with renal impairment had wounds that healed in 20.2 days, whereas those without renal impairment had wounds that healed in 17.8 days. On average, wound healing required 20.3 days for patients with hyperuricemia and 16.55 days for those without it. Patients who had undergone lower extremity surgery healed in 18.9 days, whereas those who had undergone upper extremity surgery had wounds that healed in 13.1 days. Notably, patients who had undergone surgery involving both upper and lower extremities had wounds that healed in 18.3 days.

The statistical analysis revealed that a preoperative infection (*p* < 0.0001) and lower extremity surgery (*p* < 0.016) significantly extended the LOS and wound healing time, thereby compromising surgical outcomes.

## Discussion

In patients with chronic tophus lesions, conventional surgery is typically performed only after the development of an infection or ulceration. However, patients undergoing conventional surgery under such conditions often have prolonged LOS and delayed wound healing 12. Conversely, surgery involving the shaver technique is performed upon patient request; early intervention is a key advantage of shaver-assisted surgery.

Wang et al. 18 compared two groups of patients—one underwent arthroscopic surgery and received oral medication, whereas the other received only oral medication—and concluded that the surgical group exhibited faster recovery and lower postoperative complication rates than the medication-only group 13. The shaver technique, an improved version of the arthroscopic technique, incorporates an irrigation system that minimizes the risk of obstruction during surgery. Furthermore, the shaver technique requires only 15-20 min per lesion, which is significantly less than the average time required for the arthroscopic technique (90 min). This efficiency is partly due to the use of warm saline irrigation, which increases the solubility of tophaceous materials, thereby reducing the risk of shaver tip obstruction and shortening the duration of surgery. Finally, through the integration of a shaver and manual curettage, the shaver technique allows for the removal of tophus firmly attached to the cartilage without damaging the ligament.

As shown in Tables 1 and 3, patients with a preoperative infection were more likely to have longer LOS and wound healing time. This finding lends support to the benefits of early intervention as advocated by the shaver technique. At our outpatient clinics, antibiotic treatments are initiated when patients present with signs of infection or pus from an open tophaceous wound; in addition, pus cultures are prescribed. Of the 303 procedures included in the present study, 32 (10.6%) yielded positive pus cultures: methicillin-resistant *Staphylococcus aureus* (n = 5), *Staphylococcus aureus* (n = 16), *Pseudomonas aeruginosa* (n = 6), mixed infections (n = 4; multiple bacteria: *Escherichia coli*, *Enterobacter cloacae*, *Acinetobacter baumannii* complex, and *Bacteroides fragilis*), and Group G beta-hemolytic streptococci (n = 2).

Given that blood circulation is better in the upper extremities than in lower extremities, patients undergoing upper extremity surgery tend to have relatively short LOS and wound healing time 7,8. This observation is corroborated by our study.

Our data revealed that tophaceous lesions are particularly common in the lower extremities of male patients. This trend indicates the role of estrogen in enhancing renal tubular urate excretion and thus reducing the risks of hyperuricemia and gout in premenopausal women 14. Furthermore, patients who underwent lower extremity surgery and had a preoperative infection exhibited significantly extended LOS (*p* = 0.0016) and wound healing time (*p* = 0.004). This finding further emphasizes the importance of early surgical intervention for preventing complications.

Researchers have explored the correlation between obesity and wound healing and found that individuals with obesity frequently have longer LOS, regardless of the presence of obesity-related conditions or comorbidities 15,16. However, in our patients undergoing shaver-assisted surgery, no significant correlation was observed between obesity and prolonged LOS or between obesity and delayed wound healing. This finding suggests that patients with obesity and tophaceous lesions can benefit from shaver-assisted surgery rather than conventional surgery.

Factors such as age, sex, and renal function can influence wound healing: older individuals, men, and individuals with impaired renal function often experience delayed wound healing 17,18. However, our findings indicate that these factors do not affect surgical outcomes in patients undergoing shaver-assisted surgery. Therefore, this technique may outperform conventional surgery in achieving improved surgical outcomes.

Conventional surgery typically involves the creation of a 2-to-3-cm skin incision to remove subcutaneous tophus stones; this can adversely affect the microcirculation of the area. Patients undergoing conventional surgery for gout, particularly those with factors that impede wound healing—for example, chronic inflammation, renal insufficiency, obesity, and senility—often exhibit poor wound healing and additional postoperative complications. By contrast, the shaver technique involves the creation of a 5-mm incision and the scraping of tophus lesions from the subcutaneous layer while largely preserving the subdermal plexus. The relatively small incision and improved circulation from the subdermal plexus contribute to rapid wound healing in patients undergoing shaver-assisted surgery. In our study, the shaver technique reduced the rate of postoperative complications and increased favorable wound healing outcomes, even in patients typically expected to have poor wound healing outcomes.

Emerging regenerative medicine approaches have been proven to be effective in improving the postoperative wound healing process. For example, extracellular vesicles from adipose-derived stem cells 19, platelet-rich plasma 20,21, and collaborative efforts across multiple disciplines have advanced novel wound healing methods 22. The cytokines released by adipose-derived stem cells or platelet-rich plasma can accelerate wound healing, thereby reducing LOS and optimizing shaver-assisted surgery.

This study has some limitations. The sample size differed between the patient groups. Over the 16-year study period, many patients were lost to follow-up; this reduced the availability of data for analysis. For example, in the analysis of surgical sites, LOS data were available for only 288 procedures, including 133 lower extremity procedures. However, wound healing time data were available for only 191 procedures, including 67 lower extremity procedures. As shown in Tables 2 and 4, patients undergoing lower extremity surgery were, on average, hospitalized for 19.36 days but healed in 18.92 days. This finding suggests that patients undergoing lower extremity surgery often heal completely before discharge. This discrepancy was noted in cases where patients had prolonged LOS but data on wound healing time were lost or in cases where stitches were removed before discharge because of extended LOS. To overcome this limitation, we intend to study additional cases and increase the sample size in the future.

## Conclusion

The current standard treatment for gout is ULT 23-25, which often fails to sufficiently regulate uric acid levels because of noncompliance and other challenges. Given that conventional surgery carries a risk of skin necrosis, shaver-assisted surgery should be promoted as a safe and rapid alternative to reduce the load of uric acid in the body. By emphasizing early intervention before infection onset, the shaver technique effectively addresses gout before the occurrence of joint damage, thereby improving prognosis. Furthermore, this technique minimizes the risks of wound edge skin necrosis and joint exposure—common complications of conventional surgery—and is suitable for use in patients with obesity, older patients, or patients with impaired kidney function. The preemptive approach advocated by the shaver technique has proven effective in reducing LOS. Thus, patients with chronic tophus lesions should be treated before the damage of the joint cartilage and the development of skin ulcers. Integrating the shaver technique with ULT may help optimize surgical outcomes of tophus removal, thereby improving the quality of life in patients with gout.

## Figures and Tables

**Table 1:** Mean hospitalization data on different patient parameters

Factor	Group	Number of patients	Mean hospitalization (days)		P
**Infection**	WBC<10000/ul	186	8.56		0.001
	WBC≥10000/ul	90	12.67		
**Infection**	CRP< 5	15	10.28		0.003
	CRP≥5	71	14.82		
**Renal function**	Serum Cr.<1.3mg/dl	156	9.13		0.079
	Serum Cr.≥1.3mg/dl	106	11.11		
**Hyperuricemia**	Serum UA<7 mg/dl	47	10.04		0.599
	Serum UA≥7mg/dl	131	10.76		
**Gout Attack**	Yes	52	11.56	Mean UA:8.39	0.449
	No	88	10.51	Mean UA: 8.02	
**Gender**	Male	272	9.77		0.353
	Female	15	4.04		
**Age** (years old)	30-50	134	9.20		0.201
	50-70	121	10.70		
**Age** (years old)	30-50	134	9.20		0.775
	70-90	23	8.13		
**Age** (years old)	50-70	121	10.70		0.239
	70-90	23	8.13		
**BMI** (kg/m^2^)	16-24	92	9.70		0.359
	25-30	50	8.30		
**BMI** (kg/m^2^)	16-24	92	9.70		0.441
	>30	35	8.36		
**BMI** (kg/m^2^)	25-30	50	8.30		0.999
	>30	35	8.36		

**Table 2:** Surgical site and mean hospitalization days of patients

Factor	Group	Number of patients		Mean hospitalization (days)		P
**Operation site**	Upper extremities	51		12.68		0.01
	Lower extremities	133		19.36		
**Operation site**	Upper extremities	51		12.68		0.016
	Arm + leg	104		18.04		
**Operation site**	Lower extremities	133		19.36		0.774
	Arm + leg	104		18.04		
		Infection	No infection	Infection	No infection	
**Operation site**	Upper extremities	9	25	8.00	7.68	0.877
	Lower extremities	24	56	11.92	7.88	0.016
	Arm + leg	13	56	8.82	7.69	0.097

**Table 3:** Mean wound healing time data on different patient parameters

Factor	Group	Number of patients	Wound healing(days)		P
**Infection**	WBC<10000/ul	140	16.30		0.010
	WBC≥10000/ul	47	22.74		
**Infection**	CRP<5	15	15.93		0.955
	CRP>5	29	25.79		
**Renal function**	Serum Cr.<1.3mg/dl	113	17.75		0.345
	Serum Cr.≥1.3mg/dl	63	20.19		
**Hyperuricemia**	Serum UA<7 mg/dl	31	16.55		0.319
	Serum UA≥7mg/dl	90	20.31		
**Gout Attack**	Yes	19	20.78	Mean UA:8.38	0.310
	No	48	13.27	Mean UA: 8.03	
**Gender**	Male	185	17.40		0.890
	Female	11	18.45		
**Age** (years old)	30-50	88	16.55		0.251
	50-70	86	20.34		
**Age** (years old)	30-50	88	16.55		0.545
	70-90	19	20.74		
**Age** (years old)	50-70	86	20.34		0.995
	70-90	19	20.74		
**BMI** (kg/m^2^)	16-24	73	21.62		0.834
	25-30	35	18.91		
**BMI** (kg/m^2^)	16-24	73	21.62		0.823
	>30	35	18.69		
**BMI** (kg/m^2^)	25-30	35	18.91		1.00
	>30	35	18.69		

**Table 4:** Surgical site and mean wound healing time of patients

Factor	Group	Number of patients		Wound healing (days)			P
**Operation site**	Upper extremities	39		13.05			0.017
	Lower extremities	85		18.92			
**Operation site**	Upper extremities	39		13.05			0.05
	Arm + leg	67		18.27			
**Operation site**	Lower extremities	67		18.92			0.930
	Arm + leg	85		18.27			
		Infection	No infection	Infection		No infection	
**Operation site**	Upper extremities	9	25	11.9	15.08		0.214
	Lower extremities	24	56	31.38	17.27		0.004
	Arm + leg	13	56	17.32	15.46		0.599
							
